# The Molecular Basis of Human IgG-Mediated Enhancement of C4b-Binding Protein Recruitment to Group A Streptococcus

**DOI:** 10.3389/fimmu.2019.01230

**Published:** 2019-06-04

**Authors:** David Ermert, Maisem Laabei, Antonin Weckel, Matthias Mörgelin, Martin Lundqvist, Lars Björck, Sanjay Ram, Sara Linse, Anna M. Blom

**Affiliations:** ^1^Division of Medical Protein Chemistry, Department of Translational Medicine, Lund University, Malmö, Sweden; ^2^Division of Infectious Diseases and Immunology, Department of Medicine, University of Massachusetts Medical School, Worcester, MA, United States; ^3^Colzyx AB, Lund, Sweden; ^4^Department of Biochemistry and Structural Biology, Center for Molecular Protein Science, Lund University, Lund, Sweden; ^5^Division of Infection Medicine, Department of Clinical Sciences, Lund University, Lund, Sweden

**Keywords:** the complement system, *Streptococcus pyogenes* (*S. pyogenes*), immunoglobulin G (IgG), infectious disease, protein complex, protein stability, protein-protein interaction, stoichiometry

## Abstract

*Streptococcus pyogenes* infects over 700 million people worldwide annually. Immune evasion strategies employed by the bacteria include binding of the complement inhibitors, C4b-binding protein (C4BP) and Factor H in a human-specific manner. We recently showed that human IgG increased C4BP binding to the bacterial surface, which promoted streptococcal immune evasion and increased mortality in mice. We sought to identify how IgG promotes C4BP binding to Protein H, a member of the M protein family. Dimerization of Protein H is pivotal for enhanced binding to human C4BP. First, we illustrated that Protein H, IgG, and C4BP formed a tripartite complex. Second, surface plasmon resonance revealed that Protein H binds IgG solely through Fc, but not Fab domains, and with high affinity (IgG-Protein H: K_D_ = 0.4 nM; IgG-Fc-Protein H: K_D_ ≤ 1.6 nM). Each IgG binds two Protein H molecules, while up to six molecules of Protein H bind one C4BP molecule. Third, interrupting Protein H dimerization either by raising temperature to 41°C or with a synthetic peptide prevented IgG-Protein H interactions. IgG-Fc fragments or monoclonal human IgG permitted maximal C4BP binding when used at concentrations from 0.1 to 10 mg/ml. In contrast, pooled human IgG enhanced C4BP binding at concentrations up to 1 mg/ml; decreased C4BP binding at 10 mg/ml occurred probably because of Fab-streptococcal interactions at these high IgG concentrations. Taken together, our data show how *S. pyogenes* exploits human IgG to evade complement and enhance its virulence. Elucidation of this mechanism could aid design of new therapeutics against *S. pyogenes*.

## Introduction

*Streptococcus pyogenes* is one of the clinically most important gram-positive human pathogens. The bacteria cause a spectrum of infections, ranging from mild and superficial infections to life-threatening systemic conditions (1–3). Every year, more than 700 million *S. pyogenes* infections occur worldwide with more than 500,000 deaths (4). Natural infection caused by this bacterium is restricted to humans. Group A streptococci have developed complex virulence mechanisms to avoid immune recognition and activation (5). One of the most prominent virulence factors are the surface proteins of the M protein family. M proteins have antiphagocytic properties, bind various host proteins and are involved in adherence and invasion of host cells (6–12). M proteins are also used to classify streptococci, and to date more than 200 different strain groups have been identified (13).

Protein H is a surface virulence factor that belongs to the family of M-proteins (14). It binds to a variety of human serum proteins, such as albumin, complement inhibitors including Factor H (FH) and C4b-binding protein (C4BP), and the Fc portion of immunoglobulins (8, 11, 15–17). Binding of C4BP and FH to the surface of *S. pyogenes* leads to a significant increase in virulence *in vivo* (18).

The complement system is one of the first lines of defense against invading microbes. The complement cascade comprises over 30 proteins that can mark (opsonize) targets such as pathogens, debris or foreign cells for removal by phagocytes. Complement activation also results in formation of pores that can directly lyse eukaryotic cells or gram-negative bacteria (19, 20). Since this cascade can potentially lyse host cells and cause excessive inflammation, tight control is pivotal. In addition to several membrane-bound complement inhibitors that protect the host cells from over-activation of complement, two major inhibitors regulate the cascade in solution: C4BP and FH [reviewed in (21, 22)]. C4BP inhibits the classical and the lectin pathways of complement, while FH inhibits the alternative pathway. One molecule of C4BP comprises of seven identical α-chains and a unique β-chain (23). Most interactions with ligands occur via the α-chains (22). C4BP acts as a cofactor to factor I (FI) and inactivates both soluble and cell-bound C4b, thus preventing the formation as well as accelerating the decay of the classical C3-convertase (24, 25). Unfortunately, some pathogens have evolved to hijack this host-protection mechanism [reviewed in (26)]. By coating themselves with complement inhibitors, microbes limit complement activation and evade immune recognition, thus enabling them to cause invasive infections (27).

Similar to M proteins, Protein H has a dimeric coiled-coil structure (28, 29). The binding of host serum proteins is dependent on the dimeric state of the protein, which is influenced by temperature and the presence of certain ligands such as IgG (28, 30). Recently, we showed that C4BP binding to *S. pyogenes* is enhanced by human IgG (31). Increased C4BP binding mediated by IgG resulted in decreased phagocytic elimination of bacteria and a dramatic increase in mortality in experimental streptococcal infections in mice. The molecular basis of IgG enhancement of C4BP binding remains unclear. In this study we focused on protein-protein interaction between human IgG, C4BP, and Protein H. We characterize the complex formation between the three proteins. Furthermore, we describe the influence of temperature on the molecular structure of Protein H, the effect on intramolecular dimerization and the implications for ligand binding, bacterial interaction and complement evasion.

Contrary to the conventional opsonic and protective functions of IgG against bacteria, here, we elucidate the molecular basis whereby IgG enhances C4BP binding to Protein H, which paradoxically enhances Group A streptococcal virulence.

## Materials and Methods

### Bacteria and Culture Conditions

*Streptococcus pyogenes* AP1 (from the WHO Collaborating Center for Reference and Research on Streptococci, Prague, Czech Republic) and its isogenic mutant BMJ71 lacking Protein H and M protein (32) was grown in Todd-Hewitt broth (Oxoid) at 37°C if not stated otherwise and 5% CO_2_ overnight. To ensure maximal expression of virulence factors, overnight cultures were subsequently diluted to OD_600_ = 0.1 in fresh Todd-Hewitt broth and further incubated at 37°C in 5% CO_2_ to an OD_600_ of 0.3–0.4. Prior to use, bacteria were washed with PBS and adjusted to desired OD_600_.

### Proteins and Antibodies

Fab fragments of pooled human IgG were purchased from Calbiochem, pooled human Fc fragments from Athens Research, human IgG (IVIG; Kiovig) from Baxalta. Immunoglobulins were purchased from Sigma (IgA, IgM, IgG3, and IgG4), Amgen (denosumab), Athens Research and Technology (IgG1, IgG2), Abcam (IgD), Thermo Fisher (IgE), and goat control IgG from Molecular Innovations. Denosumab (Amgen) is a monoclonal hu-IgG2 antibody that recognizes human RANKL and is used to treat osteoporosis, but does not recognize *S. pyogenes* (31). For flow cytometric analysis, the following Abs were used: mouse anti–hu-C4BP MK104 (33) coupled to biotin, goat anti human IgG (H+L)−647 (Invitrogen). Streptavidin PE (eBioscience) was used to detect MK104-biotin. Human C4BP was purified from human plasma; Protein H was expressed and purified from *E. coli*, all according to previously described protocols (11, 34). The AHP peptide was a gift from Prof. Lars Björck (35). α1-antitrypsin [α1AT; a gift of the late Prof. Bertil Laurell (36)] was used as a negative control for binding experiments. Plasma purified C4BP preparations of 2 mg/ml contained between 2 and 10 μg/ml human IgG, as determined by a sandwich ELISA for human IgG.

### Radioactive Labeling of Proteins With ^125^Iodine

Purified proteins C4BP or Protein H (40 μg) were labeled with each 18.5 MBq (Hartmann Analytics) in the presence of 2 iodobeads (Pierce, Thermo Fisher) for 15 min according to the manufacturer's instruction. Free radioactivity was removed using a PD-10 gel filtration column (GE Healthcare) and eluted with 500 μl PBS. Fractions containing the majority of radioactivity (usually 3) were pooled and used for experiments. For storage at −20°C 500 μl glycerol was added. Specific activity was measured by analyzing 2 μl of the glycerol stock in a Wizard^2^ gamma counter (Perkin Elmer). ^125^I-C4BP had an average specific activity of about 30.000 cpm/μl, while ^125^I-Protein H had in average 75.000 cpm/μl.

### Binding of ^125^I- Protein H, ^125^I-C4BP to Purified Proteins or Bacteria

Purified proteins (C4BP, IgA, IgD, IgE, IgG, IgM, IgG1, IgG2, IgG3, and IgG4) were diluted to 5 μg/ml in PBS and immobilized onto microtiter plates (Maxisorp breakapart, Nunc) at 4°C overnight. The plates were washed 3 times with wash buffer (50 mM Tris pH 8.0, 150 mM NaCl, 0.1% Tween 20) and non-specific binding sites were blocked with 3% fish gelatin (Norland Products) in wash buffer. ^125^I- labeled Protein H (50 kcpm) was diluted in binding-PBS (PBS supplemented with 0.1% Tween-20 and 0.1% BSA) and added to the immobilized proteins; in case of immobilized C4BP, indicated amounts of IVIG were added as stated in the individual experiments. After overnight incubation at 4°C and subsequent washing, using a Wizard^2^ gamma counter (Perkin Elmer) radioactivity in the wells was measured. ^125^I- labeled C4BP (500 Kcpm) was added to 5^*^10^5^ bacteria in a final volume of 100 μl in the presence of indicated amounts of different IgG preparations and incubated at 37°C for 1 h. Bacteria were washed thrice with PBS prior to counting bacteria-associated radioactivity using a Wizard^2^ gamma counter (Perkin Elmer).

### Complement Deposition and IgG Binding Assays

Bacteria (1^*^10^6^/sample) were incubated in 100 μl PBS containing 10% normal human serum (NHS), 20 μg purified C4BP or 10% human (hu-) C4BP transgenic (tg) mouse serum in the presence or absence of 200 nmol of the synthetic peptide QKQQQLETEKQISEASRKS (AHP) for 30 min at 37°C in 5% CO_2_. Bacteria were washed thrice with PBS before and after each staining step. Bacteria were subsequently stained to detect surface-bound IgG and C4BP with either anti-C4BP or anti-human IgG, respectively. Bacteria were visualized by staining with 1 μM cell trace violet (Invitrogen). Samples were analyzed using a Cytoflex flow cytometer (Beckman Coulter). Negative controls included unstained bacteria as well as bacteria stained only with streptavidin-PE.

### Native Protein Agarose Gel Electrophoresis

To form protein complexes, C4BP, IgG, and Protein H were mixed in a molar ratio of 1:10:10 in PBS for 1 h at 37°C prior to loading. All proteins in different lanes were loaded in same quantity, either directly loaded onto gels (single proteins) or pre-incubated together in PBS for 1 h at 37°C (for all mixtures). Agarose-gel electrophoresis was run for 3–4 h at pH 8.6 in 75 mM-sodium barbital buffer on a flexible polyester film Gelbond TM as described previously (37). Subsequently, the fixed agarose gel was stained with Coomassie blue.

### Nanoparticle Tracking Analysis (NTA)

NTA is used to study complex formation of proteins. NTA records the scattered light from objects in solution, where after the NTA software tracks the individual scattered lights over time. By using the Stokes-Einstein equation the size of the individual objects can be determined (38). IgG (130 nM), C4BP (130 nM), and Protein H (800 nM) were mixed and injected into the sample cell. The sample was monitored using a NanoSight LM10 HS, equipped with a 405 nm laser; 30 s videos were collected at different time points and the data analyzed using NTA software.

### Electron Microscopy

The presence and location of individual molecules was analyzed by negative staining electron microscopy, as described previously (39).To visualize the aggregation of C4BP and protein H (molar ratio 1:10), both proteins were co-incubated in the presence or absence of 1 mg/ml IVIG for 1 h at 37°C. To analyze self-assembly, 10 μg of Protein H was incubated either at 4 or 37°C before adsorbtion to the grids. The grids were rendered hydrophilic by glow discharge at low pressure in air. Five microliter aliquots were adsorbed onto carbon-coated grids for 1 min. After washing with two drops of water, the samples were stained with two drops of 0.75% uranyl-formate. Specimens were examined using a Philips/FEI CM 100 electron microscope operated at an accelerating voltage of 80 kV; images were recorded with an OSIS Veleta, side-mounted digital slow scan 2 k × 2 k CCD camera system using DigitalMicrograph™ software. The area of protein complexes and the length of Protein H molecules was measured in Adobe Photoshop CS6. Proteins, which were in closer contact than 30 nm or less, were considered to interact or to be colocalized.

### Complex Formation for Macroscopic Analysis

C4BP (0.15 nM) was mixed with IgG (0.75 nM) and Protein H (1.5 nM) in a break-apart 8 well-strip. As controls, combinations of only two proteins were also prepared as indicated. Pictures were taken at room temperature immediately after combining the proteins without any incubation time.

### Size Exclusion Chromatography

All size exclusion chromatography was performed using an Äkta explorer system (GE Healthcare) employing a Superose 6 10/30 column using PBS as eluent; 0.6 ml/min flow at ambient temperature. Proteins were incubated together in PBS for 30 min at room temperature at indicated ratios before analysis or left untreated as single protein controls. Individual proteins or protein mixtures were injected in 200 μl PBS and the absorbance at 280 nm subtracted by 320 nm (indicated as Abs_280−320nm_) was recorded to identify the elution profile. To analyze Protein H and AHP interaction absorbance 215 nm was measured since the peptide absorbs only weakly at 280 nm. The binding stoichiometry and KD were estimated by fitting equation 1 to the data

$$\begin{array}{l} \text{A}=\varepsilon \;\left(\text{0.5}\left({\text{C}}_{\text{L}}{\text{-C}}_{\text{B}}{\text{-K}}_{\text{D}}\right)+\surd \left(\text{0.25}{\left({\text{C}}_{\text{L}}{\text{-C}}_{\text{B}}{\text{-K}}_{\text{D}}\right)}^{2}{\text{+C}}_{\text{L}}{\text{K}}_{\text{D}}\right)\right) \end{array}$$

where A is the observed integrated absorbance of the peak corresponding to the free form of the varied proteins, C_L_, the total concentration of the varied protein, C_B_, the total concentration of binding sites on the constant protein, and ε the extinction coefficient. The stoichiometry was obtained as the value of the ratio CB/CP that produced the lowest error square sum, where CP is the total concentration of the constant protein. The size exclusion column was calibrated using the high molecular weight (HMW) gel filtration calibration kit (GE Healthcare) to estimate the molecular weight by size exclusion.

### Surface Plasmon Resonance (SPR)

All SPR experiments were conducted on a Biacore 3,000 system (GE Healthcare). IgG was immobilized by injecting 30 μg/ml IgG in 10 mM sodium acetate pH 5.1 buffer at a flow rate of 5 μl/min, onto channel 2, 3, and 4 of an EDC/NHS activated CM5 chip (GE Healthcare). Channel 1 served as an EDC/NHS activated blank channel. All four channels of the chip were blocked with ethanolamine before affinity measurements. Association and dissociation measurements were performed in 10 mM Hepes/NaOH, 150 mM NaCl, 3.4 mM EDTA, 0.005% Tween 20 (HBS-EP) buffer (GE Healthcare) at 30 μl/min flow rate. The chip was regenerated with 300 μl 10 mM glycine/HCl pH 2.7 between runs. Protein H was diluted in HBS-EP buffer (GE Healthcare). A measurement with 10 h dissociation time was used for k_off_ determination. Experiments with different concentrations of Protein H were set up with short dissociation times in between experiments for k_on_ determination. BIAevaluation Software was used to fit the data to 1:1 Langmuir kinetics using the mode “Kinetics Separate kon/koff.” The SPR for IgG-Fc were conducted in similar way except that in order not overestimate KD the fitting was done with a fixed value of koff (1^*^10^−4^ s^−1^), since the obtained values for koff spread over 3 orders of magnitude for individual experiments (2.6^*^10^−4^−3.8^*^10^−7^ s^−1^).

### Thermophoresis

Because C4BP interactions are difficult to analyze using SPR due to avidity effects (6–7 identical α-chains), we also performed analysis by thermophoresis. The thermophoresis instrument, Monolith NT.115, was used for the K_D_ determination. Protein H was labeled with Alexa-488 succinimidyl ester in phosphate buffer followed by gel filtration to remove free dye. All experiments were conducted in MST buffer (50 mM Tris-HCl pH 7.4, 150 mM NaCl, 10 mM Mg Cl_2_, 0.05 % Tween-20). Samples contained Protein H and C4BP. Protein H concentration was kept constant at 0.5 nM, while C4BP was used at 16 different concentrations. The measurements were conducted at 37°C, 100% LED power, and 80 or 100 % MST power. Equation 2 was fitted to the data:

$$\begin{array}{l} \text{S}={\text{S1 + S2}}^{*}\left[\text{L}\right]/\left(\left[\text{L}\right]\text{+KD}\right) \end{array}$$

Where S1 is the thermophoresis signal for free Protein H, S2 is the signal for the complex, and [L] = (0.5(C_L_-C_H_-K_D_)+√(0.25(C_L_-C_H_-K_D_)^2^+C_L_K_D_)), where C_L_ is the total concentration of the protein being varied, and C_H_, the total concentration of Protein H.

### Calculating Stoichiometry

To determine stoichiometry of the C4BP-IgG-protein complex, we premixed C4BP and Protein H (Figures 4A,C,E) or Protein H and IgG (Figures 4B,D,F) in different molar ratios. The concentration of one reaction partner was always kept constant while the amount of the other component was increased gradually as indicated. For every sample (indicated with different colors in the elution diagram) we performed SEC to identify the unbound amount of either Protein H (Figure 4A) or IgG (Figure 4D). AUC of the peaks of unbound protein were then plotted against the total amount of that protein (Figures 4B,E). This resulted in a binding curve allowing us to calculate a KD value as well as the binding sites for the indicated proteins.

### Aggregation of Bacteria

Bacterial aggregation was measured according to a previously published protocol (35) with the following modifications: bacteria were grown overnight at 30, 35, 37, and 41°C in 13 ml sample tubes filled with 10 ml Todd Hewitt broth. Immediately after removing the tubes form the incubator, bacteria were gently shaken (Vortex Genie 2, intensity 2, 3 s) and OD_600_ was measured. To assess total amount of bacteria and exclude growth differences, samples were subsequently vortexed (vortex, intensity 10, 10 s) and OD_600_ was measured (WPA spectrophotometer). To calculate % bacterial dispersion, OD_600_ before vortexing was divided by OD_600nm_ after vortexing and multiplied by 100 ([OD_600nm_ before vortexing/OD_600nm_ after vortexing] ^*^ 100). To assess growth differences due to temperature, we compared OD_600nm_ after vortexing and found that all bacteria showed identical ODs in all tested temperatures after vortexing. Analysis of IgG binding to bacteria grown at different temperatures showed that Protein H was ubiquitously expressed.

### Serum Preparation

Human serum was prepared according to (40). Human blood was allowed to clot for 30 min before centrifuged two times for 7 min at 700 × g. Serum was aliquoted and stored at −80°C. Mouse serum was prepared as described before (18). Animals were anesthetized with Isoflurane, and blood was collected by cardiac heart puncture. Blood samples were kept on ice for 30 min and allowed to clot before centrifuging for 10 min at 1,700 g and 4°C. Serum was separated, aliquoted, and frozen immediately at −80°C until use.

### Statistical Analysis

Statistical analysis was performed using GraphPad Prism 7.0b. To test for significance, we used 1-way or 2-way ANOVA analysis with Bonferroni's or Tukey's post-test tests or student's *t*-test as indicated. *P* < 0.05 was considered to be significant.

## Results

**Figure 1:**
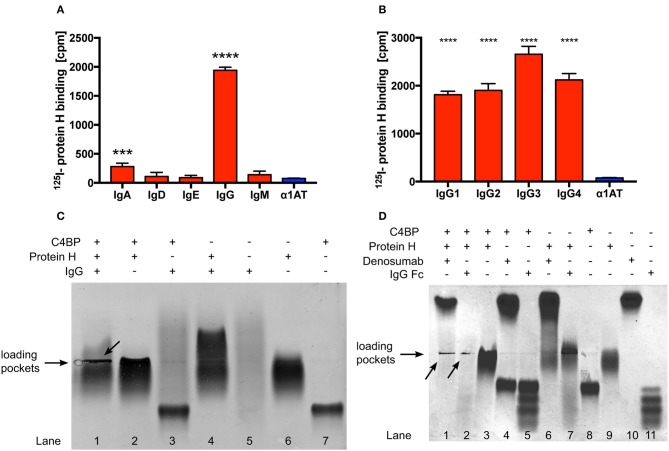
Protein H binds human IgG and C4BP. Protein H binds to IgG and to a minor extent also IgA, but not other classes of immunoglobulins **(A)**. All four tested subclasses of human IgG bind Protein H in a similar manner with a preference for IgG3 **(B)**. Protein H, C4BP and IgG, alone or in combinations **(C)**, as well as Denosumab and IgG-Fc together with C4BP and Protein H **(D)** were separated on a native horizontal agarose gel and stained with Coomassie blue. Arrows indicate protein complexes which did not migrate. The mean (SD) of three separate experiments is shown in **(A)** and **(B)**. One representative experiment of at least three repeats is shown in **(C)** and **(D)**. *** *p* < 0.001 and **** *p* < 0.0001 assessed by 1-way ANOVA **(A)** and **(B)** with Bonferroni's post-test compared to α1 anti-trypsin (α1AT), an irrelevant protein control.

Protein H is a surface bound virulence factor known to bind the Fc region of human IgG. In addition to IgG, Protein H also binds to C4BP. Surprisingly, despite overlapping binding sites for IgG and C4BP on Protein H, we observed that human IgG increased binding of C4BP to Protein H, protecting *S. pyogenes* from immune recognition and clearance (31). However, the details of the molecular interactions between Protein H, IgG and C4BP are not completely understood. Using ^125^-I labeled Protein H we confirmed that IgG is the most prominent class of Ig that binds to Protein H. We detected low amounts of IgA binding, but no significant binding of the remaining Ig classes (Figure 1A). In addition, Protein H bound to all four sub-classes of IgG. We noted similar binding to IgG1, IgG2, and IgG4, while slightly higher binding to IgG3 was observed (Figure 1B; *p* < 0.0001 compared to IgG1/IgG2; *p* = 0.0004 compared to IgG4).

In order to further define if IgG interacted with Protein H and/or C4BP, we performed native protein agarose gel electrophoresis, which does not disrupt protein-protein complexes. Proteins are separated according to their native charge, so migration can occur in either direction. Proteins may appear as a distinct band (as in the case of C4BP; Figure 1C, lane 7) or as a diffuse smear in the case of IgG (Figure 1C, lane 5) because (polyclonal) human IgG (IVIG) is a heterogenous mixture of molecules derived from >1,000 donors, each with a unique pI and glycosylation pattern. The following combinations of proteins were separated: Protein H with IgG; Protein H with C4BP; C4BP with IgG, and all the three proteins together. When compared with the migration pattern of the proteins individually (Figure 1C; lanes 5, 6, and 7), agarose electrophoresis revealed that IgG interacted with Protein H but not with C4BP (lanes 4 and 3, respectively). Further, C4BP interacted with Protein H alone (lane 2). As expected, IgG formed a complex with Protein H and C4BP, which resulted in large complexes that were retained in loading pockets (lane 1). Migration patterns of protein complexes differ from proteins migrating individually due to changes in form, size, and charge, which led to the conclusion that Protein H interacts both with C4BP and IgG, however C4BP does not interact with IgG. We repeated this experiment using IgG-Fc fragments as well as a monoclonal human IgG2 (Denosumab) to reduce the influence of Fab mediated binding to Protein H from pooled human IgG (Figure 1D). Differences were particularly obvious in lanes 10 and 11 (IgG-Fc and Denosumab) comparing the “only antibody” lanes the to the IVIG lane in Figure 1C lane 5. IgG-Fc fragments are derived from pooled human IgG, thus the four distinct bands may reflect different IgG subclasses and/or different Fc glycosylation patterns. In contrast, lane 10 shows a distinct band instead of a smear. This is because Denosumab is a recombinantly expressed monoclonal IgG2 antibody with a single glycosylation pattern. We used Denosumab as a source of a recombinant human monoclonal antibody that does not recognize *S. pyogenes*. The interactions between IgG and Protein H as well as C4BP and Protein H are almost identical. Protein H interacts with both IgG-Fc and denosumab (lanes 6 and 7), C4BP does not bind to IgG-Fc or Denosumab (lanes 4 and 5) but to Protein H (lane 3). All three proteins together form an insoluble precipitate in the loading pocket (lanes 1 and 2; indicated by arrows) as already seen with IVIG in Figure 1C. The distinct band in the upper part of lane 1 Figure 1D is attributed to an excess of Denosumab. Of note, this band is less prominent compared to lane 10 despite the same amounts of IgG loaded. A similar band for IgG-Fc does not appear in lane 2, because we used the same molar equivalent of IgG-Fc as intact IgG; Fc is only a third of the mass of IgG, resulting in a signal that is about two-thirds less intense. Taken together, we found similar interaction patterns with Protein H and C4BP and IgG independent on the type of IgG (polyclonal, monoclonal or IgG-Fc) used. Thus, our data shows that IgG-Fc mediates the increased C4BP binding to Protein H (Figure 1D).

### Protein H Forms Large Complexes With C4BP and IgG

**Figure 2:**
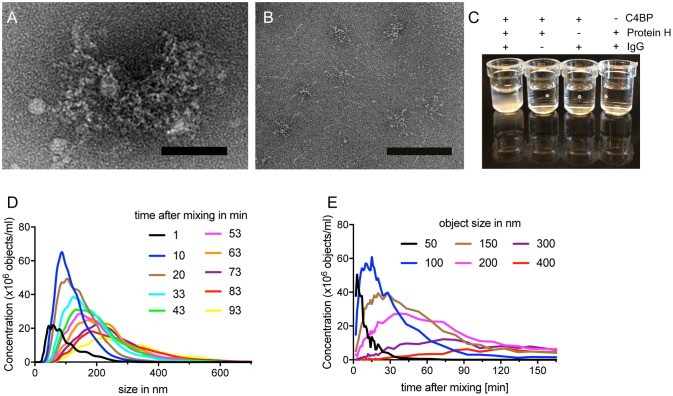
C4BP, Protein H and IgG form large complexes *in vitro*. **(A)** Electron microscopy images of negative stained protein complexes formed between IVIG, Protein H and C4BP or only C4BP and Protein H **(B)**. **(C)** Protein H together with C4BP and IgG forms large complexes in solution, which are visible macroscopically. C4BP, IgG and Protein H were co-incubated for indicated time and analyzed by nanoparticle tracking analysis (NTA) to estimate complex size at a given time **(D)** or the concentration change of object size over time **(E)**. Bars indicate 50 nm. Representative experiments of at least 3 repeats are shown.

We observed, that Protein H together with IgG and C4BP, formed complexes which remained in the loading pockets of the agarose gel (arrows in Figures 1C–D). We sought to analyze this complex further employing electron microscopy. The analysis showed large tripartite complexes of Protein H, IgG, and C4BP covering up to 35,000 nm^2^ (Figure 2A). In the absence of IgG, C4BP and Protein H complexes covered a maximal area of 2,400 nm^2^ (Figure 2B). All three proteins (molar ratio of C4BP:Protein H:IgG 1:10:5) together formed macroscopically visible aggregates (Figure 2C, first well) while any combination of only 2 proteins showed no visible precipitation. To further analyze the complex between protein H, IgG and C4BP we performed nanoparticle-tracking analysis (NTA). We found that the concentration of larger particles increased over time, while the numbers of smaller complexes decreased (Figure 2D). Particle size analysis showed that smaller particles rapidly decreased in concentration, while larger particles increase over time and finally exceeded the limit of analysis (Figure 2E). Control experiments with NTA shows that any other combination of proteins, at the concentration and ratio used in this experiment does not form large aggregates (Figure S1). C4BP alone or in combination with either IgG or Protein H is stable and does not show any changes in size or concentration over a time period of 1.5 h (Figures S1A–C). IgG alone does not scatter enough light to be detected with NTA (Figure S1D), and does not form large aggregates in the presence of Protein H (Figure S1E).

**Figure 3:**
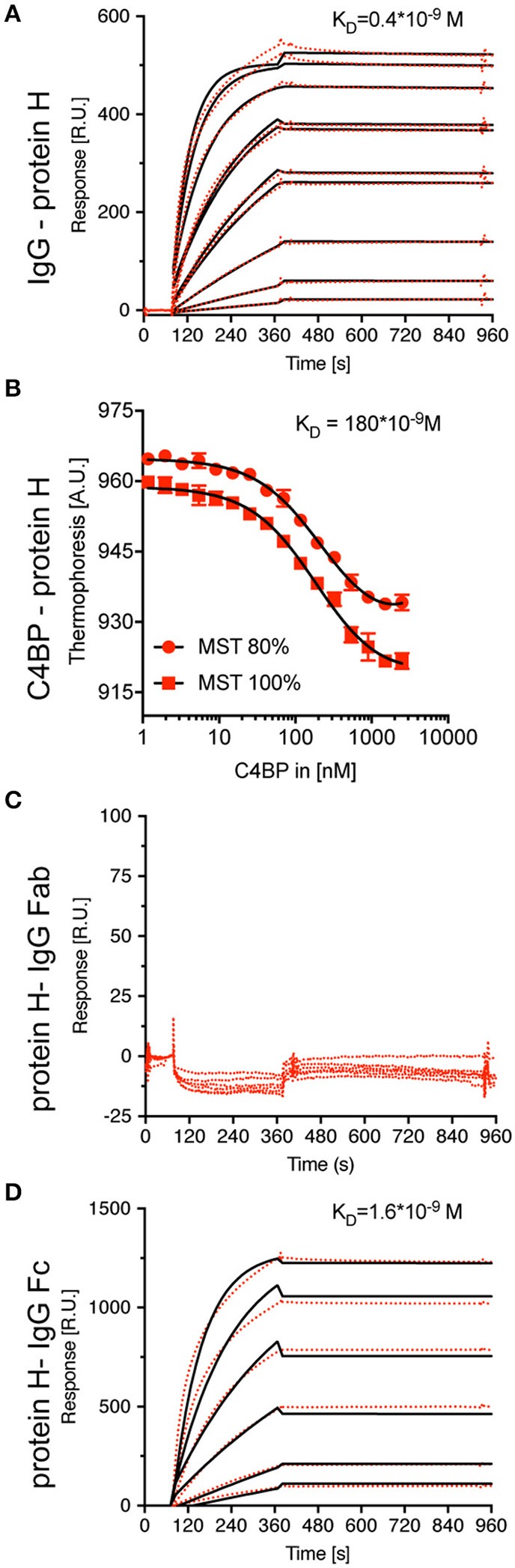
Protein H binds C4BP and IgG (-Fc) with high affinity, but not IgG-Fab. To define equilibrium affinity constants for IgG-Protein H **(A)** and C4BP-Protein H **(B)** interaction, surface plasmon resonance and thermophoresis analysis were performed, respectively. Surface plasmon resonance analysis revealed that Protein H does not interact with IgG-Fab **(C)** but binds IgG-Fc strongly **(D)**. Red dots represent individual measurements, black lines are corresponding fits.

We then aimed to determine the K_D_ values for the interaction of IgG-Protein H and C4BP-Protein H. Surface plasmon resonance analysis of IgG-Protein H revealed a strong interaction with a K_D_ value of 0.4 nM (Figure 3A). C4BP is made of 7 identical α-chains each of which contains potential binding sites for Protein H. We applied thermophoresis to estimate the affinity between Protein H and C4BP, which revealed a K_D_ of 180 nM (Figure 3B). Protein H did not interact with IgG Fab fragments (Figure 3C). Protein H bound to Fc fragments derived from pooled IgG with a K_D_ of < 1.6 nM, which was similar to the interaction between Protein H and intact IgG (Figure 3D). This indicates that IgG in human serum recognizes Protein H on *S. pyogenes* through its Fc domain.

### Stoichiometry of C4BP-Protein H and Protein H-IgG

**Figure 4:**
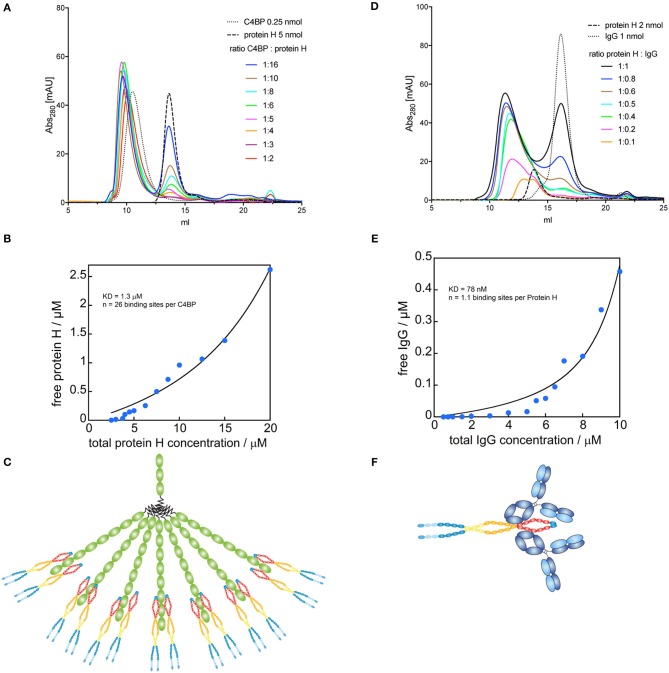
Binding sites for Protein H on C4BP and IgG. C4BP-Protein H and Protein H-IgG interactions were analyzed in combination by SEC on a Superose 6 column. **(A)** C4BP-Protein H complexes were formed in PBS with 0.25 nmol C4BP and varying amounts of Protein H (indicated by molar ratio). **(B)** AUC of free Protein H (eluted at 13.74 ml) was plotted against total Protein H concentration (*n* = 13). **(C)** Analysis revealed that one molecule of C4BP can bind up to 26 ± 2 Protein H monomers and is shown diagrammatically. **(D)** Protein H-IgG complexes were formed in PBS with 2 nmol Protein H and varying amounts of IgG (indicated by molar ratio). **(E)** AUC of free IgG (eluted at 16.19 ml) was plotted against total IgG concentration (*n* = 15). **(F)** Analysis revealed that one Protein H dimer can bind up to two IgG and is represented diagrammatically. In **(B,D)** blue dots represent individual measurements, black lines are corresponding fits. Every curve or data point represents an independent experiment. In **(A,D)** representative elution curves are displayed, while all experiments were used for the analysis in **(B,E)**.

Since C4BP has several potential binding sites for Protein H, we sought to identify the stoichiometry of the binding between Protein H and C4BP as well as Protein H and IgG. We performed size exclusion chromatography (SEC) of C4BP-Protein H mixtures at different molar ratios (Figure 4A). Subsequent analysis of free Protein H revealed the stoichiometry of the interaction (Figure 4B): one molecule C4BP can bind up to 26 ± 2 Protein H monomers. At room temperature, presumably two Protein H dimers bind to one α-chain of C4BP (Figure 4C). Similarly, we analyzed the Protein H-IgG complex by SEC (Figure 4D). Analysis of free Protein H showed that one IgG molecule bound a single monomer of Protein H (Figure 4E). We therefore speculate that a Protein H dimer could bind two IgGs (Figure 4F).

### Temperature Influence on Protein H Dimerization

**Figure 5:**
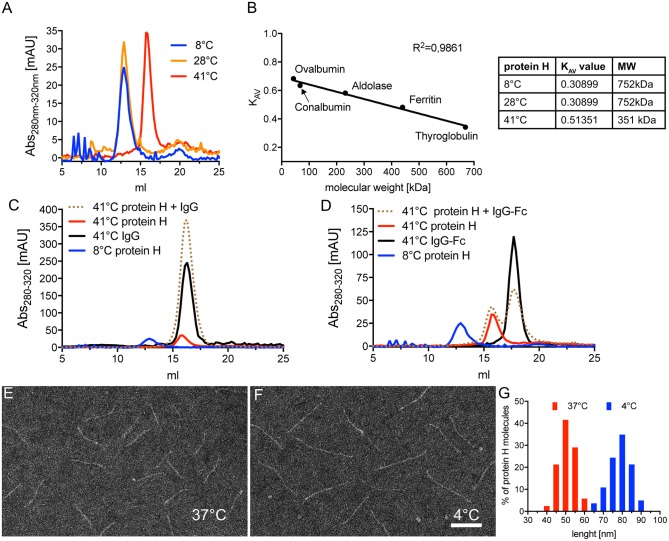
Protein H dimerization is temperature dependent and crucial for IgG binding. **(A)** Size exclusion chromatography (SEC) analysis of 5 nmol Protein H at different temperatures using a Superose 6 10/30 column. **(B)** Calibration of SEC on a Superose6 10/30 column using high molecular weight marker proteins to allow estimation of protein sizes. For size estimation of mono- and dimers of Protein H, KAV values were calculated using the formula KAV = (Ve-Vo)/(Vt-Vo) with Ve = elution volume in ml; Vo = void volume (estimated with blue dextran 2,000 = 8.67 ml) and Vt = total column volume (estimated by salt peak = 22.36 ml). **(C,D)** SEC analysis of 5 nM Protein H with equimolar amounts of IgG's at different temperatures using a Superose6 10/30 column. Protein H does not bind denosumab **(C)** or pooled IgG-Fc **(D)** at 41°C. As a reference for Protein H dimers, 4°C run of Protein H was inserted into both diagrams. Electron microscopy images of negative stained Protein H at 37°C **(E)** and 4°C **(F)**. **(G)** Size distribution of Protein H molecules at different temperatures. Representative experiments of at least 3 independent repetitions are shown. In **(E–G)** more than 500 molecules for each temperature from different areas on several microscopy grids were analyzed. Bar in **(F)** indicates 50 nm.

To assess the influence of temperature on Protein H we performed SEC on Protein H at different temperatures. At 8°C and 28°C we found that Protein H eluted at 12.9 ml, while at 41°C protein eluted later at 15.9 ml (Figure 5A). Because larger proteins elute earlier, Protein H appeared to elute as a dimer at 8°C and 28°C, but as a monomer at 41°C. To confirm this, we calibrated the column using standard marker proteins and calculated K_AV_ values to estimate the molecular weight of Protein H (Figure 5B). At lower temperatures, Protein H appeared to have a size of ≈750 kDa, while at 41°C the size estimate was ≈350 kDa, confirming the elution as dimers and monomers, respectively (Figure 5B, table). The apparent discrepancy of theoretical or calculated (42 kDa) and observed MW (350 kDa) of monomeric Protein H is because of the elongated shape of the protein. Globular proteins are used for calibration, while Protein H has a cylindrical, needle-like shape. Yet, an increase in size by a factor of two in purified preparations of Protein H strongly suggests the formation of a dimer.

We then sought to determine whether dimerization of Protein H also affected IgG binding. Using SEC analysis, we found that monomeric Protein H at 41°C did not bind IgG (Figure 5C). The elution curve of Protein H incubated with IgG overlapped with the elution curves of IgG or Protein H alone. As a control, the elution curve of dimerized Protein H at 8°C has been superimposed. Similarly, binding of IgG-Fc to monomeric Protein H did not occur at 41°C (Figure 5D); elution curves for the single proteins and the co-incubated samples were similar. Taken together, these data show that only dimeric, but not monomeric Protein H binds human IgG. Electron microscopy analysis of isolated Protein H showed an increase in length of individual Protein H molecules with a decrease in temperature. At 37°C (Figure 5E) Protein H appeared to be shorter than at 4°C (Figure 5F). Size evaluation of more than 500 molecules showed that Protein H has an average length of 50 nm at 37°C, but about 80 nm at 4°C (Figure 5G). Because the interaction site for Protein H is not located at the N-terminal tip of the protein but within the first third of the protein, dimerization did not double the length, but was less than the sum of the two proteins. This supports the notion that two Protein H molecules interact with each other in an antiparallel orientation, since a parallel interaction would not lead to an increase in length.

**Figure 6:**
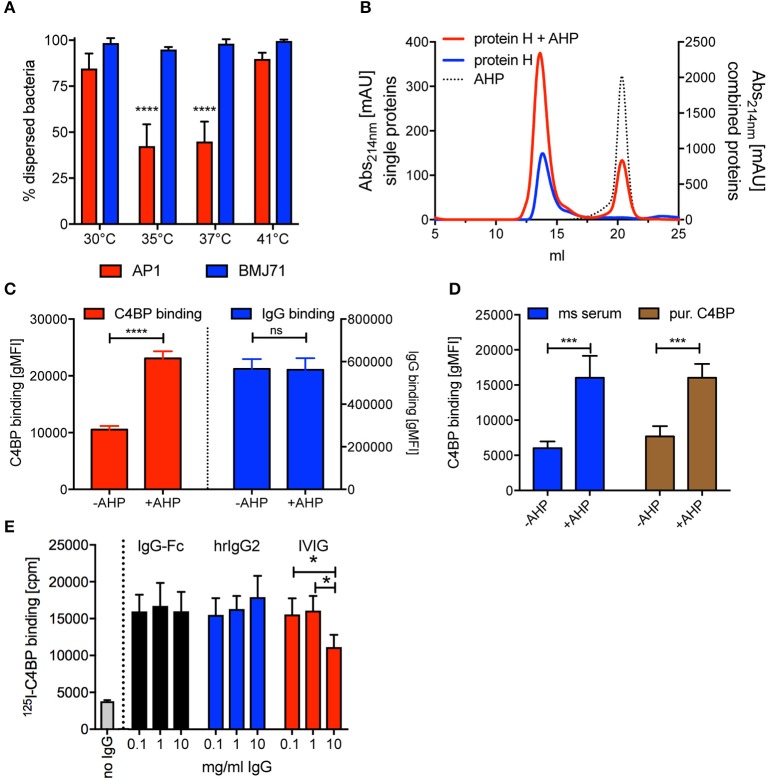
AHP does not disrupt dimers formed by purified soluble Protein H but increases C4BP binding to intact bacteria. **(A)** *S. pyogenes* AP1 grown at different temperatures showed aggregation at 35°C and 37°C. Bacterial dispersion is significantly decreased for AP1 (red bars) at 35°C compared with 30°C and 41°C as well as at 37°C compared with 30°C and 41°C. Isogenic mutant BMJ71 lacking Protein H (blue bars) shows similar absorption at all temperatures and does not aggregate. **(B)** SEC analysis using a Superose 6 10/30 column was performed on 10 nmol Protein H in the presence or absence of 100 nmol of a dimer inhibiting peptide (AHP). AHP does not prevent dimerization of Protein H. **(C)** AHP (100 nmol) increased C4BP binding to 5*10^5^ *S. pyogenes* significantly without altering IgG binding levels. **(D)** C4BP binding to *S. pyogenes* is increased in the presence of 100 nmol AHP both in mouse (ms) serum and for purified C4BP. **(E)** Presence of IgG-Fc or monoclonal human IgG2 increases ^125^I-labeled C4BP binding to Protein H independent of IgG concentration, while increasing concentrations of IVIG beyond 1 mg/ml decreases C4BP binding. Representative experiments of at least 3 repeats are shown. *p < 0.05, ***p < 0.001, and ****p < 0.0001 assessed by a 2-way ANOVA with Tukey's post-test **(A,D,E)** or a student's t test **(C)**. Comparison of significance in **(A)** was performed for different temperatures within a strain (blue bars and red bars separately).

Since Protein H-Protein H interaction is supposed to be pivotal for adherence to other bacteria causing aggregation and increased virulence (35), we asked if temperature had a similar effect on bacterial aggregation. We inoculated Todd Hewitt broth with *S. pyogenes* AP1 and incubated it overnight either at 35°C, 37°C, or 41°C. We then measured the OD_600_ of the medium and compared it to the OD_600_ after vortexing the cultures thoroughly ([OD_600nm_ before vortexing/OD_600nm_ after vortexing] ^*^ 100). We found that at 35°C and 37°C about 70 % of the bacteria aggregated, while at 41°C all bacteria remained dispersed (Figure 6A, red bars). Cultures of *S. pyogenes* BMJ71 that lacked Protein H showed no aggregation at all tested temperatures (Figure 6A, blue bars). Of note, the absolute OD_600nm_ after vortexing of the different cultures were similar, indicating no growth defect of the mutant strain or the wild type. IgG binding experiments performed at room temperature with wild-type bacteria grown at the before mentioned temperatures showed similar Protein H expression at all temperatures tested. Thus, the difference in aggregation was not the result of altered expression of Protein H or a difference in growth.

A previous report showed that a synthetic peptide with a sequence of QKQQQLETEKQISEASRKS (aggregative Protein H peptide 150–168; AHP) blocked binding of purified Protein H to intact bacteria and also prevented bacterial aggregation, similar to temperatures above 37°C (35). We performed SEC analysis of Protein H at room temperature (23°C, where Protein H exists as a dimer) in the presence and absence of AHP, to analyse the influence of the peptide on Protein H dimerization. Interestingly, the addition of the inhibitory peptide did not change the elution curve of Protein H even at a 10 molar excess of AHP (Figure 6B). This indicates that AHP does not interrupt dimer formation between soluble Protein H molecules.

We then incubated *S. pyogenes* AP1 in NHS in the presence and absence of AHP and analyzed C4BP and IgG binding. To our surprise, we found that in the presence of AHP C4BP binding was increased significantly by more than 2-fold while IgG binding was unaffected (Figure 6C). This effect could be due to more Protein H dimers available to bind to C4BP instead of to other Protein H dimers. To see, if complement deposition on bacteria affected C4BP binding in the presence or absence of AHP, we repeated the experiment with hu-C4BP tg mouse serum or C4BP purified from human serum (Figure 6D). In fact, AHP had a similar effect on C4BP binding to *S. pyogenes* strain AP1 independent on the source of C4BP, excluding effects from complement deposition. The presence of peptide increased C4BP binding to bacteria 2–2.5 fold.

We previously reported that C4BP binding to Protein H peaked at 1 mg/ml, but rather surprisingly, decreased at higher IgG concentrations (31). We asked if this effect was specific for pooled human IgG (IVIG) or also occurred with IgG-Fc or monoclonal human IgGs. Thus, we tested ^125^I-labeled C4BP binding to *S. pyogenes* in the presence of increasing amounts of IVIG, IgG-Fc or monoclonal human IgG. Unlike IVIG, neither IgG-Fc nor monoclonal IgG induced a decrease of ^125^-I C4BP binding to Protein H at high concentrations (10 mg/ml) (Figure 6E). Consistent with previous observations (31), we observed decreased C4BP binding to *S. pyogenes* at 10 mg/ml IVIG.

**Figure 7:**
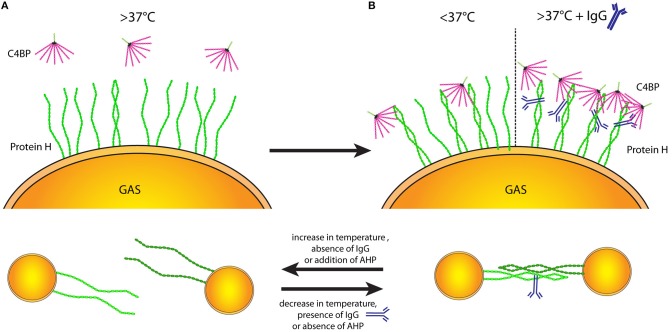
Protein H structure influences ligand binding and interbacterial interaction. Temperature-dependent dimerization allows various interactions of Protein H. **(A)** At temperatures of 37°C and above the majority of Protein H is monomerized and cannot bind any ligands (incl. C4BP). **(B)** Upon decrease of temperature, increasing numbers of Protein H homodimers form and allow interaction with other molecules (left side). Beside low temperatures, IgG binding can stabilize Protein H homodimers even at high temperatures so that Protein H retains its binding capacity to different ligands (right side). **(C)** Protein H homodimers do not only bind to ligands, but also allow homo-tetramer formation to promote interbacterial interaction. Protein H monomers however cannot facilitate bacteria-bacteria interactions. Similar to ligand binding, lower temperature and presence of IgG at high temperatures stabilize Protein H dimers allowing for tetramer formation (right side). An increase of temperature as well as absence of IgG a high temperature causes Protein H monomerization and loss of function. AHP also causes loss of protein-Protein H interaction (tetrameric state reduced to dimeric state) and can at large molar excess also cause monomerization of Protein H.

At temperatures >37°C Protein H on the bacterial surface is mainly monomeric (Figure 7A), thus unable to bind ligands. Stable Protein H dimers capable of binding its ligands occur at lower temperatures or in the presence of human IgG Fc domains (Figure 7B). We hypothesize that Protein H mediated adherence of *S. pyogenes* to human tissues or the formation of bacterial aggregates is dependent on dimerization of Protein H (Figure 7C).

## Discussion

In this study we show that C4BP can interact with Protein H and human IgG in a 1:13:26 molar ratio to form very large insoluble complexes (> 5 MDa) (Figures 1, 4). We also found that dimerization of Protein H is favored at lower temperatures (Figure 5A). Importantly, ligand binding is exclusively restricted to dimeric Protein H (Figures 5C–D). We also determined that bacterium-bacterium interactions via Protein H is temperature dependent (Figure 6C).

Recently we showed that IgG binding to Protein H enhances C4BP binding to Protein H (31). This was unexpected because C4BP and IgG occupy overlapping binding sites on Protein H and therefore one would expect a competition in binding between the two proteins rather than the observed synergistic binding (11, 15, 31). We found that human IgG-Fc specifically stabilizes the dimeric structure of Protein H and therefore allows for increased C4BP binding to the bacteria. Data presented here shed light on the molecular details of how *S. pyogenes* Protein H uses human IgG to capture more C4BP to evade complement.

C4BP is a molecule with seven identical α-chains and a unique β-chain (23). The identified binding site for M proteins as well as Protein H is localized to the N-terminus of the α-chain (11, 41, 42), which also contains the complement inhibitory site (43). C4BP can interact with up to four C4b molecules (44). Earlier, we reported that C4BP bound to *S. pyogenes* via Protein H is still functional and can indeed act as a cofactor for Factor I thus degrading C4b on the bacterial surface (11). Since C4BP can bind only up to four C4b simultaneously, the remaining three α-chains (arms) are available to interact with surface bound Protein H to confer protection to *S. pyogenes*. Testing the complex of Protein H, IgG and C4BP for cofactor activity to Factor I *in vitro* is not feasible, since the complex immediately precipitates. However, the fact that the FI-cofactor activity of C4BP is retained when bound to bacterial surfaces as well as in complexes, for example with DNA, suggests that the C4BP-IgG-Protein H complex will also have cofactor activity (45–47).

These immunocomplexes between C4BP, IgG, and Protein H may also be formed after a cleavage of Protein H from the surface of *S. pyogenes*. The bacteria express a soluble virulence factor, SpeB, which is a promiscuous cysteine protease, which releases Protein H from the surface. The 36 kDa fragment of Protein H that is released binds IgG and given the overlapping nature of the IgG and C4BP binding sites, it is also likely to bind C4BP (48). It was proposed, that shedding of Protein H and M proteins promote spreading of infection and could contribute to post-streptococcal glomerulonephritis (48, 49). The large complexes formed between the released fragment of Protein H, IgG, and C4BP could support the formation of microthrombi during streptococcal infections, one of the hallmarks of systemic streptococcal infections (50, 51).

The gene for Protein H lies adjacent to the M protein gene, and presumably has evolved by gene duplication (52). Similar to M proteins, Protein H also presents a coiled-coil dimer structure with a heptad repeat pattern (28). Protein H dimers are stabilized by low temperatures; Protein H exists as a monomer above 37°C, indicating low thermal stability (Figure 5A). This dimeric structure however seems to be a prerequisite for binding to human protein ligands. Monomeric Protein H does not only form homodimers, but it can bind itself and M1 in an antiparallel manner (28). This is believed to facilitate interactions with adjacent bacteria, or even with host cells (35). Thus, in this fashion, the Protein H structure may influence host pathogen interactions. Interestingly, addition of ligands, such as albumin or human IgG significantly increases the stability of Protein H dimers (28). Of note, IgG does not bind to purified monomeric Protein H at temperatures above 37°C, and therefore does not induce dimer formation at supra-physiologic temperatures (30).

In addition to Protein H, other streptococcal IgG binding proteins such as Arp4, Sir and M1 also bind IgG only below 37°C (30, 53). Notably, all these biochemical studies were performed with purified proteins. We recently showed that on the bacterial surface, Protein H binds IgG even at 37 and 39°C (31). This may be related to anchoring of the protein to the bacterial surface as well as the density of Protein H, or to other proximate proteins. All those factors may influence the conformation of Protein H. These variables are absent when analyzing isolated Protein H and could explain contradictory results when using purified proteins vs. proteins *in situ*.

Why C4BP binding to *S. pyogenes* AP1 peaks at IgG levels of 1 mg/ml instead of the physiological serum concentration of 10–15 mg/ml remains unclear (Figure 6E). One hypothesis is that IgG-Fc directed binding competes with Fab directed binding (of whole IgG) to Protein H. However, we previously showed that human IgG exclusively binds to Protein H via Fc domains and not through the antigen recognition sites on the antibody (Figure 1D) (31). By comparing F(ab), Fc, and whole IgG binding to Protein H, we only observed background binding to F(ab) independent on the amounts used (31). Thus, it does not appear to be a direct competition, probably due to the lack of anti-M/H protein antibodies. Considering the hypervariability of M proteins, including Protein H, it seems plausible that only very little antibodies from a pool of >1,000 people can bind isolated Protein H from *S. pyogenes* AP1 (29, 54). This hypervariability is also one of the main reasons why a broadly protective vaccine against *S. pyogenes* remains elusive (55). A more likely explanation why C4BP binding peaked at 1 mg/ml IgG is orientation of the antibody on the bacteria. It was shown that streptococci bind polyclonal human immunoglobulins predominantly via their Fc region in the presence of low IgG concentrations, but at high (e.g., 10 mg/ml) concentrations Fab binding dominates, because a small fraction of the IgG in human sera IVIG contains IgG whose Fab is directed against bacteria (56). Of note, Fab binding occurs rather to whole bacteria, but not to Protein H itself. This also explains why the human mAb denosumab did not block C4BP binding even at 10 mg/ml (Figure 6E). If the bacteria are covered with antibodies bound via the Fab domains, the Fc domains cannot interact with Protein H to stabilize the dimeric structure. That together with the thermal instability of Protein H at physiological temperatures explains the reduced C4BP binding.

Similar to IgG and C4BP binding to Protein H, we found that the interaction between bacteria is also temperature-dependent (Figure 6A). The majority of streptococcal infections are suppurative infections such as pharyngitis and pyoderma, which involve peripheral tissues with a temperature lower than the body core temperature (5, 57). Lower temperatures and a lower IgG level compared to those encountered in circulation might support the stability of the M-protein family (M protein and Protein H) and could explain streptococcal virulence especially at cutaneous or mucosal surfaces. At temperatures between 35 and 37°C bacteria aggregated due to Protein H, however at 41°C no aggregation was observed despite the presence of Protein H. This indicates, that the aggregation of *S. pyogenes* AP1 and most likely other strains as well-depend on the homophilic anti-parallel interactions between Protein H dimers and similar proteins to form a Protein H- Protein H homo-tetramer. This tetramerization could be interrupted not only through the AHP peptide (35), but also by temperature. Of note, the AHP peptide preferably binds Protein H dimers and thus interferes with tetramerization. Only at a 1,000-fold molar excess Frick et al. observed < 50% monomerization of Protein H. Another effect of AHP is the increase of C4BP binding to *S. pyogenes* (Figure 6C). The peptide interrupts Protein H dimer-dimer interactions between bacteria, but not Protein H dimer formation. We hypothesize that the peptide renders more binding sites available for C4BP, which were previously blocked by Protein H-Protein H interactions (Figure 6C).

High fevers caused by streptococcal infections may induce monomerization of Protein H/M protein, which in turn could curtail the invasive capacity of the bacteria and reduce immune evasion (58). Thus, the febrile response may help the immune system counteract this bacterial virulence mechanism and support bacterial clearance. We found that in the absence of IgG, C4BP binding decreased with increasing temperature (schematic representation in Figure 7A). Compared to 30°C, and beginning at 37°C, < 50% of C4BP binding was observed. This effect was reverted in the presence of IgG; C4BP binding to *S. pyogenes* was maximal at all measured temperatures (31). Thus, it appears that monomerization does not occur in an all-or-nothing fashion, but rather gradually peaking at 41°C with 100% of Protein H being in a monomeric form (Figure 5A). Taken together, IgG seems to counteract Protein H monomerization on *S. pyogenes* by stabilizing Protein H at higher temperatures (Figure 7B), leading to more C4BP binding and increased resistance to complement. The molecular reason might be the stabilized dimeric coiled-coil structure of Protein H preventing dissociation into monomers, thereby enabling it to retain its capacity to bind ligands.

Taken together, targeting Protein H to prevent dimerization would reduce C4BP binding and enable increased complement deposition to clear the bacteria. This could be possible at any given temperature, so that the effect of fever is not a prerequisite. Another therapeutic approach would be to target the Protein H-IgG interaction, because a lack of IgG binding would monomerize Protein H and again reduce C4BP binding—this modality would be effective in febrile conditions, where the Fc domain of IgG stabilizes Protein H dimers. That way, even bacterial aggregates would be targeted, which are important for virulence and colonization (Figure 7C) (35).

Here we provide explanation and a physiological function for IgG binding proteins, which contributes to improved survival of *S. pyogenes* and an advantage for the bacteria in the ongoing and evolving struggle between the host and pathogen.

## Contribution to the Field statement

*Streptococcus pyogenes* (GAS), an exclusively human pathogen, affects over 700 million people worldwide annually. GAS has evolved several mechanisms to avoid host immune surveillance. In one such strategy, Protein H, a member of the M protein surface adhesin family, binds human IgG via its Fc-domain, thereby subverting opsonophagocytosis. We previously showed that a second effect of Protein H-IgG Fc interactions was to enhance recruitment of the soluble complement inhibitor, C4b-binding protein (C4BP). Increased C4BP acquisition by GAS prevents efficient complement activation and reduces phagocytic clearance. Herein, we elucidate the molecular mechanism of this observation. We show that dimerization of Protein H is required for IgG Fc binding. IgG stabilizes Protein H dimers at body core temperatures and allows stable interactions with C4BP. Protein H, IgG Fc, and C4BP form a stable tripartite structure. Importantly, this phenomenon occurs at low IgG concentrations, as may be encountered in extravascular compartments. In contrast, enhanced C4BP binding is abolished when IgG Fab interacts with Protein H, as observed with pooled human IgG. This knowledge can be harnessed to develop novel treatments and may also explain the clinical utility of intravenous IgG (IVIG) therapy for certain GAS infections.

## Data Availability

All datasets generated for this study are included in the manuscript and/or the Supplementary Files.

## Ethics Statement

This study was carried out in accordance with the recommendations of the local ethical committee in Lund, Sweden (permit 2017/582) with written informed consent from all subjects in accordance with the Declaration of Helsinki. This study was carried out in accordance with the recommendations of Guide for the Care and Use of Laboratory Animals of the U.S. National Institutes of Health and the Swedish Animal Welfare Act SFS1988:534. The protocol was approved by the Institutional Animal Care and Use Committee at the University of Massachusetts Medical School or by the Laboratory Animal Ethics Committee of Malmö/Lund, Sweden.

## Author Contributions

DE and AB: Conceptualization. DE, SL, and AB: Methodology. DE, MLa, AW, MM, and MLu: Investigation. DE, SR, and AB: Writing—original draft. DE, MLa, AW, MM, LB, SR, SL, and AB: Writing—review and editing. DE, SR, and AB: Funding acquisition. DE, MM, LB, SL, SR, and AB: Resources. DE and AB: Supervision.

### Conflict of Interest Statement

The authors declare that the research was conducted in the absence of any commercial or financial relationships that could be construed as a potential conflict of interest.

## Supplementary Material

Figure S1 Nanoparticle Tracking Analysis for C4BP, IgG and Protein H alone or in combination. Panel **(A)** is the results for C4BP alone and **(B)** in combination with IgG and **(C)** in combination with Protein H. Panel **(D)** is IgG alone and **(E)** in combination with Protein H. The legend reposts the time, in min, after the sample were mixed. The concentration reported at the y-axis should be multiplied with 10^6^.
