# Supplementary figures and images for: The Molecular Basis of Human IgG-Mediated Enhancement of C4b-Binding Protein Recruitment to Group A Streptococcus
Source: Front Immunol. 2019 Jun 4;10:1230. doi: 10.3389/fimmu.2019.01230 (PMC6557989; doi:10.3389/fimmu.2019.01230)

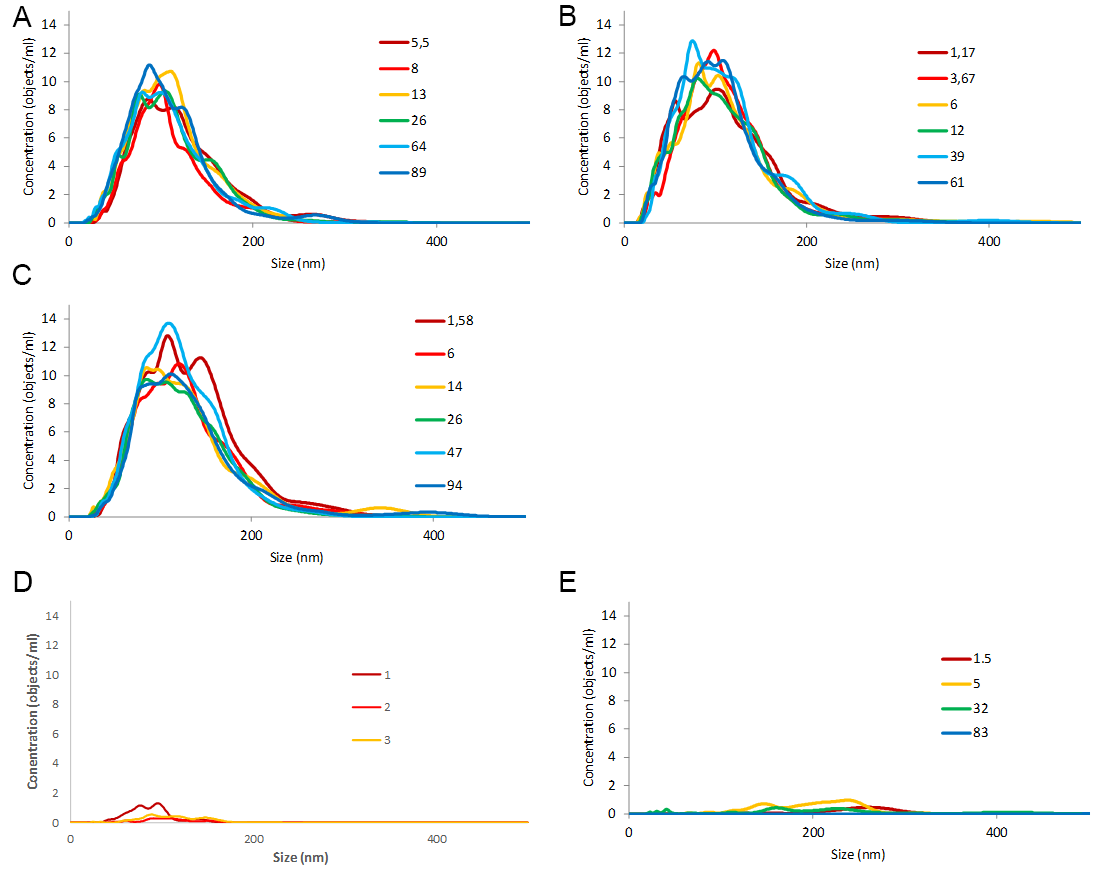

Supplement: Figure S1 — Nanoparticle Tracking Analysis for C4BP, IgG and Protein H alone or in combination. Panel (A) is the results for C4BP alone and (B) in combination with IgG and (C) in combination with Protein H. Panel (D) is IgG alone and (E) in combination with Protein H. The legend reposts the time, in min, after the sample were mixed. The concentration reported at the y-axis should be multiplied with 106. [file Image_1.tif]
